# Protective Behavioral Strategies and Alcohol Consumption: The Moderating Role of Drinking-Group Gender Composition

**DOI:** 10.3390/ijerph16050900

**Published:** 2019-03-12

**Authors:** Carmen Tabernero, Tamara Gutiérrez-Domingo, Bárbara Luque, Olaya García-Vázquez, Esther Cuadrado

**Affiliations:** 1 Department of Social Psychology, INCYL, University of Salamanca, 37007 Salamanca, Spain; 2 University of Córdoba, IMIBIC, 5000 Cordoba, Spain; tamara.gutierrez@uco.es (T.G.-D.); bluque@uco.es (B.L.); esther.cuadrado@uco.es (E.C.); 3 University of Salamanca, 37007 Salamanca, Spain; id00684062@usal.es

**Keywords:** protective behavioral strategies, alcohol consumption, group composition, gender differences, health behavior

## Abstract

**Background**. There is international concern about the negative consequences for health related to young people’s alcohol consumption. Peer relationships can play a positive and protective role to cope with risky behaviors associated with alcohol consumption. **Objective**. This study investigated the influence of protective behavioral strategies (PBS) on alcohol consumption and the moderating role of drinking-group gender composition and drinking-group size. **Methods**. The sample comprised 286 youths (mean age = 23.49; *SD* = 2.78; 67.5% female). Participants reported their protective behavioral strategies, their alcohol consumption and the size (overall mean = 7.44; *SD* = 3.83) and gender composition (62.58% mixed; 19.93% all-female; 9.8% all-male) of their social drinking groups. The mean sizes of mixed, all-female, and all-male groups were 8.27, 5.34, and 6.2, respectively. **Results**. Data showed that women consume less alcohol and use more protective strategies than men, particularly those strategies directed at avoiding negative consequences. Furthermore, the number of men in a group influences protective strategies and consumption, therefore drinking-group gender composition moderates the relationship between protective strategies and alcohol consumption. The more protective strategies that young adults use, the lower their alcohol consumption. This relationship is moderated by the size of the group. **Conclusion**. Strategies to prevent risky drinking behavior should focus on both PBS shared by drinking-group members and the training in individual PBS associated with drinking behavior. Finally, taking into account the relationship between drinking-group gender composition and protective behavioral strategies for alcohol consumption, a positive protector role for individual and group habits in relation to alcohol consumption is discussed.

## 1. Implications of Alcohol Consumption in Youth and Its Moderators

Alcohol consumption in early life is one of the biggest global problems of the last decade, especially in Western countries—although we know there are differences between regional areas in alcohol consumption and their customs [1]. The Global Status Report on Alcohol and Health published by the World Health Organization (WHO) [2] states that alcohol per capita in populations older than 15 years showed a small consumption decrease in Spain in recent years: from 9.6 L of pure alcohol on average for 2009–2011 to 8.5 L for 2015–2017 (means for the WHO European Region were 11.2 and 9.8, respectively). Globally 5.9% of all deaths—3.3 million p.a.—are caused by harmful consumption of alcohol [3]. This WHO report (2014) [3] stated that harmful consumption of alcohol is not only an important contributing cause of 5.1% of global illnesses, mental disorders and infection diseases but is also responsible for large economic and social costs. A review of qualitative studies provided by the WHO (2014) [3] concluded that young adult drinking patterns present a serious problem and that young adults lack awareness of the negative consequences of irresponsible drinking.

In recent years the scientific evidence on the consequences of binge drinking amongst young people has prompted leading authorities to act to control the growing problems caused by alcohol abuse, which has negative effects on psychological and neurological health [4]. It is well known that the harmful use of alcohol—both the quantity and the frequency—is related to truancy, bad grades, poor relationships, dependency, and legal issues [5], poor social adaptation, depression and other mental disorders [6], abuse, poor sexual health, disability, violence and many more risky behaviors [7]. The consumption of alcohol not only influences the person who drinks it but also their network of peers. Perkins (2002) [8] showed that student peers’ norms influence students’ personal drinking behavior. In addition, alcohol-related road traffic accidents are a problem that affects everyone who drives after drinking, not just young people [5]; consequently, the drink–driving laws have been hardened in recent years. Despite cultural differences, it is known that alcohol motives and patterns among young people are similar in many countries [9], which is why society is said to confront an international problem rather than a national or local one.

It is generally held that this alcohol phenomenon is associated more with college students than other young people, which suggests that it is related to university rules or culture surrounding alcohol consumption [10]. Excessive expectations, lack of autonomy, elevated student support and low student satisfaction have been shown to influence alcohol consumption [1]. Impulsivity, sensation-seeking, sensitivity to anxiety, hopelessness, extraversion and lack of awareness are characteristics of personality related to binge drinking [11]. Furthermore, there are other psychosocial factors that influence alcohol consumption, for example, family alcohol habits associated with the age of onset of consumption [12], or drinking alcohol expectancies and cultural norms [13]. Research suggests that there are different motives or reasons for alcohol consumption [14] but, as Pearson (2013) [12] noted, there is no consensus about the predictive role of motivational variables or personal traits on alcohol consumption, so more research needs to be done on how the social network influences alcohol consumption or substance use. Thus, the aim of this study was to investigate psychosocial factors that might have an impact on alcohol consumption in young people. More specifically, we studied the protective influence of behavioral strategies developed to control alcohol consumption and the effects of social group size and drinking-group gender composition on alcohol consumption.

### 1.1. Protective Behavioral Strategies Related to Alcohol Consumption

Protective behavioral strategies (PBS) are specific behaviors used to minimize the harmful consequences of alcohol consumption [12]. Some authors [15] have indicated that PBS can reduce—and in some cases prevent—the consequences of alcohol consumption and, hence, alcohol-related problems. For example, Neilson, Gilmore, Pinsky, Shepard, Lewis, and George (2018) [16] have shown the important role of both drinking and sexual assault PBS in the prevention of sexual victimization. Furthermore, Gilmore et al. [17] have shown that those females who suffered sexual attacks had used less PBS than those who did not have negative experiences of sexual assault. Similarly, Benton et al. (2004) [15] reported that individuals who use PBS suffer fewer negative consequences of alcohol consumption even if they drink a lot; however, this study received a mixed reception and it was argued that there were problems with the methodology and sampling strategy [18]. It is legitimate to emphasize the importance of investigating how PBS could be taught to young people as a tool to reduce binge drinking, alcohol consumption and negative drinking consequences.

In this sense, a comparative study found that most teens reported drinking for social motives (to improve social interaction) and some reported enhancement motives (drinking to enhance mood) but only a small proportion reported coping motives (drinking to ameliorate negative mood) [19]. Following this argument, LaBrie and colleagues [20] showed that PBS use mediates the relationship between “drinking motives” and “alcohol consumption”, and this was also corroborated by considering gender differences.

### 1.2. Gender Differences in Alcohol Consumption and Use of PBS

In this section we discuss the relationship between PBS and gender. The gender differences in alcohol consumption in young people are quite surprising. One of the largest sociodemographic studies [21] showed that marriage decreased alcohol consumption in both genders, employment decreased alcohol consumption in young women but increased it in young men and parenthood is associated with a reduction in the frequency of drinking amongst women. Although males report greater alcohol consumption than females, young women report greater use of PBS [20]. A recent meta-analysis of PBS concluded that women tend to use more PBS than men (Pearson, 2013). Although young women are less likely to experience harmful consequences of alcohol, they consume smaller quantities of alcoholic beverages and they are more likely to use PBS than their male peers. Further investigations have studied the network of relationships linking PBS, mental health and gender; PBS use was shown to protect females, regardless of mental health [22].

In summary, this study focuses on gender differences in alcohol consumption and how the use of specific PBS would be higher amongst young women than young men. This research sought to shed light on how gender is related to the amount of alcohol consumption and the use of PBS to reduce consumption. Although earlier age of alcohol consumption and binge drinking amongst young women have increased [23], we hypothesized that male college students would still display greater and more frequent consumption than their female peers. In other words, we expected to find that young men are still drinking more than young women.

### 1.3. Alcohol Consumption as a Social Act in Young Adults

Previous studies [24,25] have shown that peers have a great influence on the drinking habits of their reference group, and that this influence is particularly strong in the case of young adults. In this sense, Moses and Villodas (2017) [26] have found that peers’ companionship relationship can play a positive and protective role in coping with the adversities of at-risk youths. In addition, when groups have and share a greater use of PBS, group members have fewer problems associated with risk-taking [27].

In relation to drinking-group gender and size composition, Thrul, Labhart, and Kuntsche (2017) [28] showed that young women and men consumed more drinks per hour when drinking in mixed-gender groups, whereas drinking-group size only had a predictive effect for the greater number of drinks for women. Thrul et al. (2017) [28] therefore highlighted that: group gender composition and size are associated with alcohol consumption in young adults; the greater the number of people present while consuming alcohol, the greater the amount of alcohol ingested; and the impact is greater for men than for women [29]. Thus, along with support from previous studies, we can hypothesize that per-individual consumption would be higher in groups where male youths were the majority [28].

Continuing with the problem of youth alcohol consumption, it is important to mention the importance of descriptive PBS norms. The drinking norms of a group influence group members’ use of PBS. There are links between context-specific norms, group size and compliance to the group [21], such as compliance to norms about alcohol consumption in the group (norms and habits that emerge when drinking in groups). Consequently, the influence of PBS on “alcohol consumption style” is moderated by group composition—the number of males in a group influences the total alcohol consumed in the group [28]—but PBS use still remains strongly associated with alcohol consumption (Martens et al., 2011). PBS use was shown to be negatively associated with weekly alcohol consumption and experience of negative consequences of alcohol consumption [30]. Moreover, PBS use is negatively related to alcohol consumption because it is considered as a moderator of the relationship between certain risk factors linked to alcohol abuse and alcohol outcomes [31]. The results of earlier research have been promising but they highlight the need for continued study of factors related to alcohol consumption in order to reach clear conclusions.

Our research examines two potential moderators of the relationship between “PBS use” and “alcohol consumption”: (i) the number of people in the group they usually drink with, that is, the drinking-group size; and (ii) the drinking-group gender composition, that is, the number of male and female college students in the group they usually drink with. The size and composition of one’s social group have a huge influence on adolescents; for example, it has been demonstrated that PBS use is more frequent in same-sex student groups (groups composed of only one gender) relative to gender norms—expectations related to gender [30].

### 1.4. Hypotheses

The present study has pointed out gender differences and patterns in alcohol consumption among young people. In this document, we address four research questions that attempt to clarify gender alcohol patterns. First, we examine how alcohol consumption varies with gender in a sample of young adults and why women continue to drink less than their male peers, despite the fact that, overall, inter-gender differences are now much smaller than they used to be (H1). Second, we describe how the number and frequency of use of PBS vary by gender; it is thought that women use more PBS than their male peers (H2). Third, we would like to demonstrate the encouraging effect of “gender group composition”: how male groups are more prone to have larger alcohol consumption than female groups; and how males in mixed groups have an impact (talking about greater consumption) on other males and females (H3). Lastly, we predicted that in young people the use of PBS would be negatively related to alcohol consumption and that the protective impact of PBS would be smaller in large groups than in small groups, and also smaller in groups with a higher proportion of males. In contrast, we predicted that the number of women in the group would not moderate the PBS–alcohol consumption link (H4).

## 2. Method

### 2.1. Participants

The sample comprised 286 young people. Individual questionnaires were administered to assess beliefs and behaviors associated with lifestyle behaviors related to alcohol consumption. We collected sociodemographic data: age, gender, current occupation, and level of education. The study dealt with alcohol consumption in young adulthood: the sample comprised current college and non-college students (ex-college) with a mean age of 23.49 years (*SD* = 2.78) who were Spanish or living in Spain at the time of data collection. The drinking-group gender composition of the sample was 65.7% (*n* = 188) female and 34.3% (*n* = 98) male.

### 2.2. Procedure

Before beginning the research, the ethics committee of the University of Córdoba (Comité de Bioética y Bioseguridad) approved the research procedure for the main aim of the study: “A multilevel analysis of lifestyle and drinking patterns at individual and peer levels: An explicative model to create educative programs based on evidence to reduce alcohol consumption considering a gender perspective”.

We used snowball sampling to obtain a more diverse sample of 286 young people than would have been possible if we had relied on recruiting all participants directly. The first round of data collection commenced with the recruitment of 58 educational psychology students from the University of Córdoba (Spain), who distributed the questionnaires to four or five friends they socialized with. The majority of the sample was made up of university students (72.9%) and 16.9% were not involved in academic study or not working at the time of being interviewed. Around 10% of the sample failed to answer the employment-educational situation. The students who participated were rewarded with course credits and were also informed about the confidentiality of the data.

### 2.3. Measures

*PBS related to alcohol consumption.* We used a questionnaire developed by Martens et al. (2007) to evaluate the use of PBS in our sample. A double translation procedure was used to create the Spanish version. Responses were given using a six-point Likert scale ranging from 1 = *Never* to 6 = *Always*. The questionnaire is composed of 18 items that cover many aspects of alcohol consumption habits amongst young people. The total reliability of the 18 items and the system was high (α = 0.85).

The 18 items can be organized into three types of PBS based on goal: strategies for limiting/stopping drinking, manner of drinking strategies and strategies for reducing serious negative consequences of consumption were the dependent variables. The PBS scale developed by Martens et al. (2007) [18] was used and a confirmatory factor analysis was performed to verify the three-factor structure proposed originally (X^2^ (df = 116) = 300.83; *p* < 0.001; comparative fit index (CFI) = 0.84; root-mean-square error of approximation (RMSEA) = 0.07 (low = 0.06; high = 0.08)). Conceptual inspection of the data indicated that the first factor (strategies for limiting/stopping drinking) was composed of five items (e.g., *Determine not to exceed a set number of drinks*), all of which had good reliability (α = 0.76). The second factor—strategies for manner of drinking (e.g., *Avoiding mixing different types of alcohol*)—also had high reliability (α = 0.72). The final factor—strategies for reducing serious negative consequences (e.g., *Use a designated driver*) had acceptable reliability (α = 0.65); note that this value is better than the reliability reported for this factor in the original study (α = 0.59).

*Drinking-group composition: size and gender.* The moderating variables are the size of the group and the drinking-group gender composition. All participants reported the size of the group with which they spend their leisure time (hereafter “social group”). Mean (*M*) social group size was 7.44 (*SD* = 3.83; range = 2–30), mean number of men per social group was 3.52 (*SD* = 3.28; range = 0–20) and mean number of women per social group was 3.94 (*SD* = 2.20; range = 0–11). There were 28 men who usually partied in all-male groups and 57 women who usually partied in all-female groups, but the majority of the sample (179) belonged to mixed social groups (*M* = 8.27; *SD* = 4.14). The mean size of the all-male groups was 6.21 (*SD* = 3.04) and the mean size of the all-female groups was 5.34 (*SD* = 1.46).

*Alcohol consumption style.* A double translation procedure was used to create the Spanish version. The study has focused on the last three weeks in order to have recent information on alcohol consumption habits [32,33]; interviewees reported the days on which they had gone to a party and the days on which they had consumed alcohol with the next questions “Thinking about the last three weeks, use the following calendar to indicate the days on which you have gone partying (with a circle) and the days when you have drunk some alcohol (with a cross)”; they also reported either the number of drinks or the type of alcohol with the next questions “Now indicate the number of drinks with alcohol that you have taken (being 1 drink = 1 glass of wine, or 1 can/bottle of beer, or 1 liquor, or 1 gin-tonic…), and finally, indicate the type of alcohol that you have consumed under those boxes in which you have indicated having drunk”. To ensure that we all use the same measurement of alcohol consumption, we created a specific measure for one alcoholic beverage: (a) one glass of wine; (b) one bottle/can of beer; (c) one shot of spirits. We created a dependent variable termed “alcohol consumption style” that represents a combination of two other variables: number of consumption days and number of beverages (*r* = 0.77; *p* < 0.001). The number of party days over the three-week period ranged from 0 to 21 days (*M* = 4.45; *SD* = 3.27), whereas the number of consumption days ranged from 0–15 days (*M* = 3.67; *SD* = 2.86). We also know that the average number of beverages consumed over the three-week period ranged from 0–89 drinks (*M* = 13.68; *SD* = 14.55) and the type of alcohol consumed varied in different percentages: beverages with fermented alcohol (15.3%), distilled alcohol (31%), fermented alcohol and distilled alcohol (42.9%), and non-alcoholic beverages (10.7%)

### 2.4. Analytic Strategy

We calculated descriptive statistics (*M* and *SD*) and Pearson’s correlations between the study variables with the SPSS program (v. 22) (IBM SPSS Statistics V22.0., Armonk, New York, United States). Univariate analysis of variance (ANOVA) was used to assess gender differences in alcohol consumption (H1) and PBS use (including the three subtypes of strategies) (H2). To test H3 we carried out univariate ANOVA and post hoc analyses to assess differences between the three types of group gender composition (mixed; all-female; all-male). Finally, moderation analyses with 10,000 bootstrap samples and 95% bias-corrected bootstrap confidence intervals were performed using the PROCESS macro for SPSS (Hayes, 2017) to determine whether group size and group composition moderated the effect of PBS on alcohol consumption (H4); the dependent variable was PBS; group size, number of men and number of women were introduced as moderating variables; age, gender and group (the cluster to which each participant belonged) were introduced as covariates; and alcohol consumption style was introduced as the independent variable.

## 3. Results

### 3.1. Alcohol Consumption Varies with Gender (H1)

First, we examined how alcohol consumption varied with gender. Our results were consistent with the scientific literature in that we found that the young men in our sample drank more than the young women. We found no gender difference in number of party days [*F* (1264) = 0.60, n.s.; men: *M* = 4.24 days, *SD* = 0.27; women: *M* = 4.57 days, *SD* = 0.36]. Furthermore, we did not find significant differences between gender within the days of partying and alcohol consumption (*F* (1264) = 0.43, n.s.): in concrete data, the young men drunk alcohol on an average of 3.51 party days (*SD* = 2.69) whereas the women drunk alcohol on an average of 3.75 party days (*SD* = 2.96).

The results from analyses of the variable ‘number of beverages’ (*M* = 13.68, *SD* = 14.55) showed gender differences (*F* (1256) = 4.80; *p* < 0.05; eta^2^ = 0.01; potency = 0.50). Closer inspection revealed that young men drank more (beverages: *M* = 16.33) than young women (beverages: *M* = 12.22). There were gender differences in the variable alcohol consumption style (a combined variable based on number of party days, number of beverages and number of whole drinks; higher scores represent *less* healthy consumption styles): *F* (1262) = 3.84; *p* < 0.05; η^2^ = 0.01; potency = 0.50; and the young men had higher alcohol consumption style scores (*M* = 11.81; *SD* = 13.02) than the young women (*M* = 9.20; *SD* = 8.54)—in other words, young women have healthier alcohol consumption habits than their male peers.

### 3.2. PBS Use Varies with Gender (H2)

Second, we observed the relationship between the use of different PBS and gender group composition. The young women used a higher number of PBS overall (*F* (1284) = 5.40; *p* < 0.05; η^2^ = 0.02; potency = 0.64; men: *M* = 3.25, *SD* = 0.98; women: *M* = 3.51, *SD* = 0.83) and a higher number of strategies to avoid serious negative consequences (*F* (1284) = 19.30; *p* < 0.001; η^2^ = 0.06; potency = 0.99; men: *M* = 3.65, *SD* = 1.10; women *M* = 4.19, *SD* = 0.92). Use of the other two strategy types—limitation of consumption and manner of consumption—was similar [*F* (1284) = 0.01 and *F* (1284) = 1.78, respectively].

### 3.3. Alcohol Consumption Varies by Gender Group Composition (H3)

We also examined the extent to which drinking-group gender composition moderated the relationship between PBS use and alcohol consumption. We found that groups consisting of more men consumed more alcohol. All-female groups consumed less alcohol and drank less frequently; in other words, they had a healthier alcohol consumption style. Univariate analysis showed marginal group composition-related differences in consumption of alcohol (*F* (2261) = 2.47; *p* < 0.09). Post hoc analysis revealed differences between the consumption of mixed groups and all-female groups (DMS: *t*-test = 3.44; *p* < 0.05).

Preliminary analyses are shown in Table 1, including correlations between variables over the whole sample. Alcohol consumption style score was negatively related to the three PBS factors (limitation of consumption; manner of consumption; avoidance of negative consequences) and positively related to group size and number of men. In general, the use of PBS improved alcohol consumption style and our hypotheses that group size and number of men in the group would have a detrimental effect on alcohol consumption style were confirmed.

Table 2 compares the correlations between variables in young men and young women. Alcohol consumption style score was negatively correlated with the three PBS factors (limitation of consumption; manner of consumption; avoidance of negative consequences); in other words, use of PBS is associated with a healthier alcohol consumption style. In men, but not women, group size had a negative impact on alcohol consumption style (i.e., was associated with less healthy drinking habits). On the contrary, the number of men in a group has a positive impact (more consumption) on alcohol consumption style for both males and females; however, this impact is different by gender: young men in the group have more influence over other men than over women. Despite the composition differences, we showed that the younger the men in a group, the greater the per-individual alcohol consumption.

**Figure 1:**
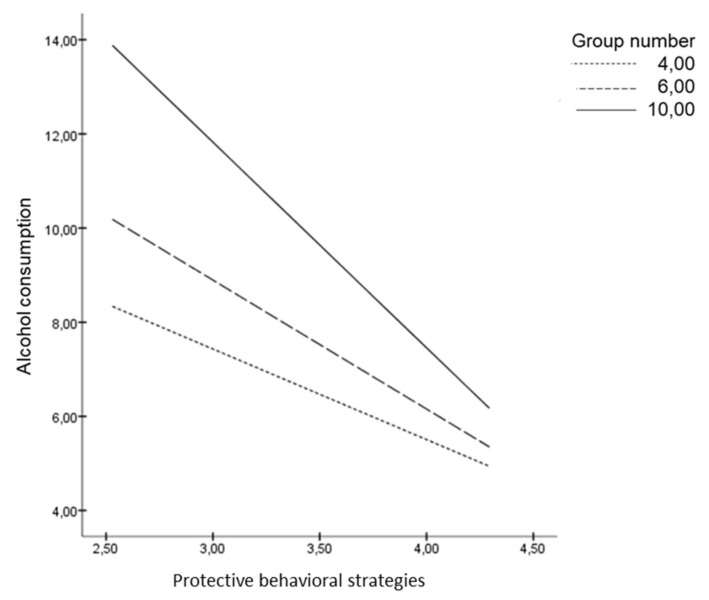
Group number as moderator of the link between PBS and alcohol consumption.

We examined the extent to which group size moderated the association between PBS and alcohol consumption style score (Figure 1). As the slope of the graph shows, group size has a negative relationship with the impact of PBS on alcohol consumption style score. The more PBS young adults use, the lower their alcohol consumption. This relationship is moderated by the size of the group, such that: (a) when young people use few PBS, group size is positively related to alcohol consumption; (b) when young people use strong PBS, group size is no longer related to alcohol consumption. These effects were controlled by including age, gender and group as covariates in the analyses.

**Figure 2:**
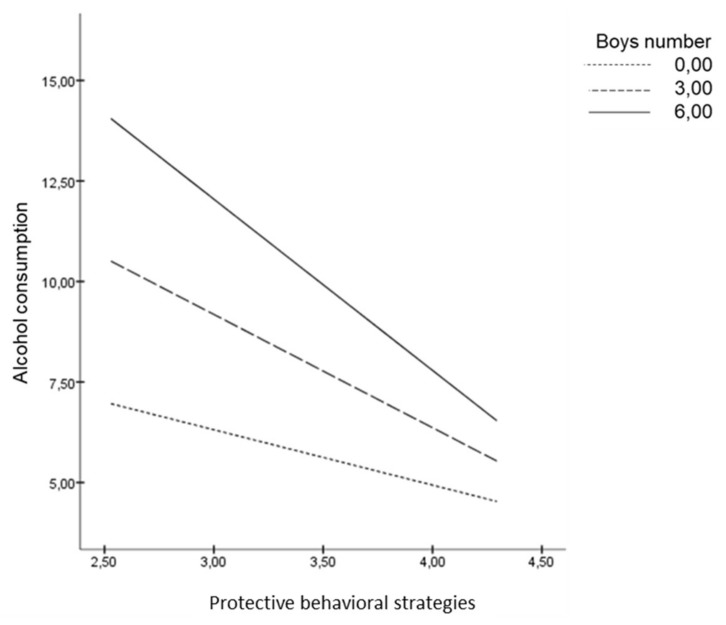
Number of men as moderator of the link between PBS and alcohol consumption.

**Figure 3:**
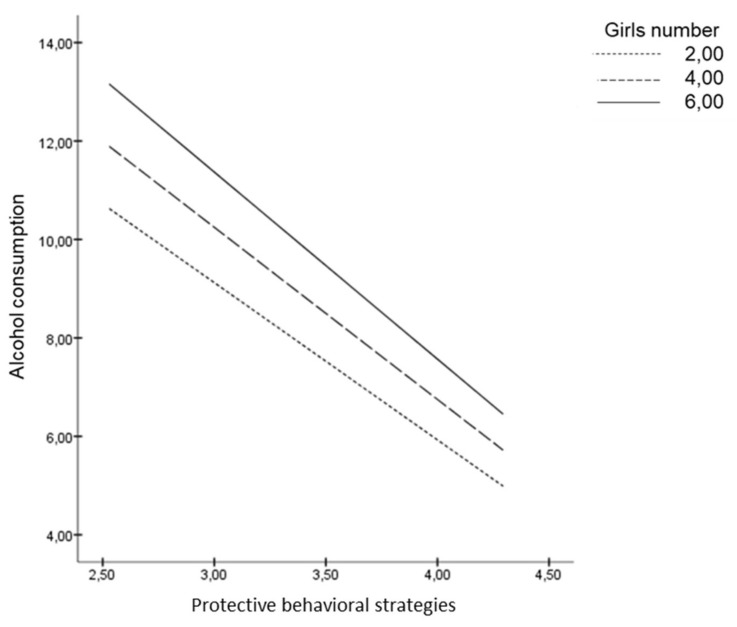
Number of women as non-moderator of the link between PBS and alcohol consumption.

If we pay attention to the moderating role of the number of men and the number of women, we can appreciate that the more men in the group, the less PBS are used. As Figure 2 shows, when young adults use few PBS, the number of men in the group is positively related to alcohol consumption, whereas when they use many PBS the alcohol consumption is independent of the number of men in a group. In contrast, as the slope of the graph shows, the number of women in a group does not moderate the relationship between PBS and alcohol consumption (Figure 3). These effects were controlled by including age, gender and group as covariates in the analyses.

The three moderation analyses are summarized in Table 3, which shows coefficients for the three moderators (group size; number of men; number of women) of the relationship between PBS and alcohol consumption.

## 4. Discussion

In this study we used a moderation model to analyze the influence of group composition (peer variables) on the relationship between PBS and alcohol consumption style. Participants were asked to complete a PBS questionnaire to provide data on the relationships between PBS and socioeconomic variables. We have offered a more detailed account of gender differences in alcohol consumption and use of PBS than earlier studies. Our results are consistent with previous studies of women’s alcohol consumption [23] and greater use of PBS relative to men [12,20]. Secondly, the study demonstrated that peer influence on alcohol consumption is different depending on the gender group composition. This study extends previous findings about the gender composition of groups [28]. Thirdly, we demonstrated the relation between PBS and alcohol consumption style, both for female and male students. Lastly, we have demonstrated that group size moderates the relationship between PBS and alcohol consumption. These findings provide additional support to consider PBS as a moderator in alcohol consumption [30,31]. Our findings have established an important framework on group composition. The importance of groups and social pressure must be kept in mind in order to explain how a group could influence its members [27] for both good and bad. These conclusions can be used to develop effective policies and interventions in this field. Practical interventions should, therefore, focus on groups and on encouraging groups of young adults to make greater use of PBS.

The social objective is to highlight the importance of having information on how behavioral patterns cluster together—and thus present as a healthy lifestyle—in young adulthood [34,35]. To more formally investigate this issue, it would be important to know how physical health is distributed in adolescence by peer group and education. The negative consequences of binge drinking are well established, and PBS are regarded as a good way of reducing alcohol consumption or minimizing the risks associated with it [31,36]. In this sense, peer relationships can play a positive and protective role in coping with the adversities [26]; and young adults who share PBS can play a positive protector role in individual and group habits regarding alcohol consumption. If we have appropriate interventions, we may be able to engineer a shift away from risky behaviors towards healthy behaviors. In the future, we are interested in investigating how both educational programs and multi-component interventions can help to minimize alcohol consumption in youth.

At the practical level, programs should be oriented towards prevention or reduction of alcohol consumption by young adults in groups. The results of our moderation analyses suggest that it would be useful to develop multi-component intervention programs (e.g., Cronce and Larimer, 2011 [35]) that train individuals to use PBS (e.g., offer a list of drinking control strategies; or training in cognitive behavioral techniques) and/or brief interventions using personalized feedback on PBS (e.g., personalized feedback interventions via email) and test them in groups of varying gender composition to determine whether group composition moderates the intervention effect. It would also be interesting to compare the effectiveness of brief individual and small-group interventions with the moderating role of drinking-group gender composition. The PBS skills training alcohol interventions with brief groups have proved effective in female college students, who are more likely to implement PBS than their male peers [36], but it appears that their effectiveness should be tested in samples of varying drinking-group gender composition. In Andalusia (south Spain) collaboration agreements are developed between the Department of Equality and Social Policies, through the General Directorate of Social Services and Attention to Drug Addiction with the nine public Andalusian Universities [37]. In this sense, studies are designed and developed with the general objective of knowing the prevalence, consumption patterns and attitudes of the university population for the planning of more effective preventive actions adapted to the consumption profile of university students. For example, in 2015, the “Activa tus sentidos” project was developed in collaboration with the Andalusian television. The purpose of this project was to design and develop an awareness and awareness campaign for the prevention of drug addiction, focusing on prevention strategies for alcohol consumption (https://www.emartv.es/proyecto_activa_tus_sentidos/).

As limitations, the snowball sampling design and the non-representative study sample are not large enough to make accurate generalizations. Due to this, it might be interesting to replicate the investigation in other environments in order to uncover potential cultural, educational, or socioeconomic differences. In this sense and following the study limitations related to taking a purely quantitative approach, future studies could be developed along qualitative lines into cultural and social aspects that would offer valuable additional insight into the drinking practices. Following this idea of future research, Larsen (2017) [38] suggests that any interventions targeted at young drinkers would have to consider the messages that this audience is likely to be receptive to: messages focused on sexual harassment might be more effective than those focused directly on alcohol harm. Another study limitation is related to statistical analysis design, regarding clusters of students in groups, now a group variable as a covariate in the analysis had been inserted; however, it is not the same as adjusting for variance attributable to group membership (i.e., hierarchical modeling), which would likely increase the size of standard errors for regression coefficients. On the other hand, although our sample was not gender balanced (65.7% female; 34.3% male), this could be considered an advantage because we were particularly interested in women’s alcohol consumption behaviors and strategies.

## 5. Conclusions

The present study has clarified several factors related to the consumption of alcohol in order to contribute to the investigation of this matter. Our main goal was to provide evidence on gender differences in alcohol consumption to inform the development of more effective policies on alcohol consumption in adolescence. We have tried to explore some of the social factors in alcohol consumption as these are currently a concern for many Western countries. Our results have demonstrated gender variations in patterns of alcohol consumptions. Women use more PBS and have a healthier alcohol consumption style than men; similarly, all-female groups use more PBS and their members have healthier alcohol consumption strategies than mixed groups and all-male groups. For all these reasons, the gender composition of social groups should be included as a variable in future studies. Many educational interventions designed to reduce alcohol consumption and empower young adults to refuse alcohol are already being deployed, but this is not enough to tackle the problems of alcohol consumption amongst young people. With this in mind, it is necessary to continue seeking ways to make educational programs more effective. To summarize, interventions should focus on encouraging groups to use PBS designed to eliminate or reduce alcohol induction and deliver responsible drinking [31].

## Figures and Tables

**Table 1:** Means, standard deviations and correlations of all variables studied.

Variable	1	2	3	4	5	6	7
1. PBS_F1 Limitation of consumption	1						
2. PBS_F2 Manner of consumption	0.58 **	1					
3. PBS_F3 Avoidance of negative consequences	0.61 **	0.41 **	1				
4. Group size	−0.11	0–0.11	−0.05	1			
5. Number of men	−0.12 *	−0.20 **	−0.14 *	0.82 **	1		
6. Number of women	−0.01	0.09	0.12 *	0.51 **	−0.07	1	
7. Alcohol consumption style score	−0.32 **	−0.38 **	−0.29 **	0.35 **	0.38 **	0.03	1
Mean	2.47	3.63	4.01	7.44	3.52	3.94	10.13
*SD*	1.14	1.09	1.02	3.83	3.29	2.21	10.41

Notes: PBS = protective behavioral strategies; *SD* = Standard deviation; (** *p* < 0.01; * *p* < 0.05).

**Table 2:** Means, standard deviations and correlations of all variables studied; male data below the diagonal and female data above.

Variables	1	2	3	4	5	6	7
1. PBS_F1 Limiting	1	0.53 **	0.57 **	−0.09	−0.09	−0.04	−0.32 **
2. PBS_F2 Manners	0.68 **	1	35 **	−0.11	−0.21 **	0.10	−0.43 **
3. PBS_F3 Consequences	0.70 **	0.50 **	1	0.01	−0.03	0.07	−0.31 **
4. Composition_number	−0.13	−0.09	−0.05	1	0.86 **	0.58 **	0.14
5. Number of men	−0.19	−0.14	−0.05	0.84 **	1	0.08	0.19 *
6. Number of women	0.03	0.02	−0.02	0.66 **	0.16	1	−0.03
7. Alcohol consumption style	−0.34 **	−0.33 **	−0.23 *	0.50 **	0.53 **	0.18	1
Men *M*	2.46	3.52	3.65	8.12	5.42	2.7	11.81
*SD*	1.24	1.09	1.10	4.64	3.51	2.52	13.03
Women *M*	2.47	3.70	4.19	7.09	2.52	4.58	9.20
*SD*	1.09	1.10	0.92	3.29	2.67	1.71	8.54

Notes: PBS = protective behavioral strategies; *SD* = Standard deviation; (** *p* < 0.0.01 * *p* < 0.05).

**Table 3:** Model coefficients for the “group composition”: group size, number of men in the group and number of women in the group. The relation Protective Behavioral Strategies—Alcohol Consumption (X) is moderated by group gender composition (M).

		Coeff.	*SE*	*t*	*p*
X (PBS)	b_1_	−0.30	1.04	−0.29	*ns*
M (group size)	b_2_	1.95	0.44	4.41	<0.001
XM (PBS x group size)	b_3_	−0.41	0.13	−3.07	<0.01
Age (covariate 1)	b_4_	0.32	0.15	2.11	<0.05
Sex (Covariate 2)	b_5_	0.01	0.09	−0.07	*ns*
Group (Covariate 3)	b_6_	−0.08	0.03	−3.20	*<0.001*
Constant	i_1_	0.34	4.95	0.07	*ns*
	*R*^2^ = 0.31 *F* (6.260) = 19.53, *p* < 0.001		Δ*R*^2^ = 0.03 *F* (1.260) = 9.42, *p* < 0.01		
X (PBS)	b_1_	−1.38	0.68	−2.01	<0.05
M (number of men)	b_2_	2.40	0.50	4.77	<0.001
XM (PBS x number of men)	b_3_	−0.48	0.15	−3.26	<0.001
Age (covariate 1)	b_4_	0.30	0.15	1.96	<0.05
Sex (Covariate 2)	b_5_	0.16	0.10	1.58	*ns*
Group (Covariate 3)	b_6_	−0.07	0.03	−2.92	*<0.01*
Constant	i_1_	5.48	4.28	1.28	*ns*
	*R*^2^ = 0.31 *F* (6.260) = 19.80, *p* < 0.001		Δ*R*^2^ = 0.03 *F* (1.260) = 10.64, *p* < 0.001		
X (PBS)	b_1_	−2.89	0.93	−3.10	<0.01
M (number of women)	b_2_	1.01	0.74	1.37	*ns*
XM (PBS x number of women)	b_3_	−0.15	0.21	−0.71	*ns*
Age (covariate 1)	b_4_	0.36	0.16	2.23	<0.05
Sex (Covariate 2)	b_5_	−0.17	0.10	−1.63	*ns*
Constant	i_1_	11.02	4.93	2.24	<0.05
	*R*^2^ = 0.22 *F* (6.260) = 11.92, *p* < 0.001		Δ*R*^2^ = 0.01 *F* (1.260) = 0.48, *ns*		

Notes: X = independent variable; M = moderator; the dependent variable is alcohol consumption style score. Coefficients are unstandardized.
